# Supplementary material for: Translational Read-Through Drugs (TRIDs) Are Able to Restore Protein Expression and Ciliogenesis in Fibroblasts of Patients with Retinitis Pigmentosa Caused by a Premature Termination Codon in FAM161A
Source: Int J Mol Sci. 2022 Mar 24;23(7):3541. doi: 10.3390/ijms23073541 (PMC8998412; doi:10.3390/ijms23073541)
Supplement: Supplementary file 1 [file ijms-23-03541-s001.zip › Supplementary Materials 170322.pdf]

Supplementary Materials:

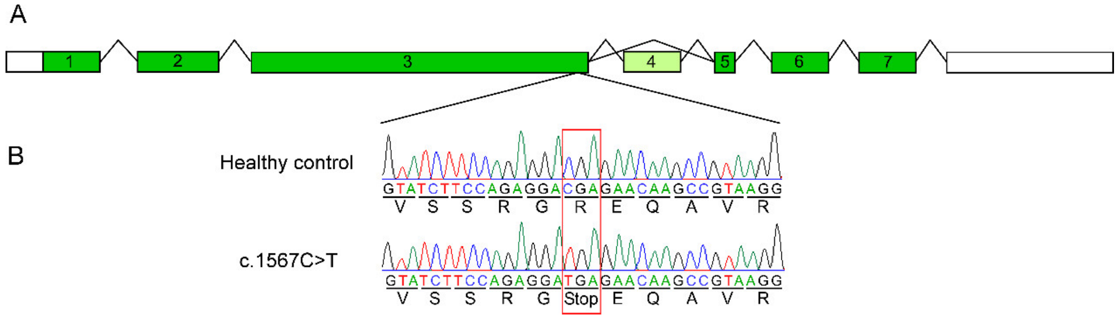

Figure S1. *FAM161A* gene structure and c.1567C>T mutation location. (**A**) A schematic representation of the human *FAM161A* gene. Green boxes represent exons 1-7, light green is the alternative exon 4 that can be spliced out, creating a short isoform. White boxes represent untranslated regions (UTRs). (**B**) The pathogenic nonsense mutation in *FAM161A* gene. Towards the end of exon 3, a point mutation causes a premature termination codon c.1567C>T (p.R523*). Representative chromatograms of a healthy control and *a* homozygous patient (MOL1300-1) are presented.

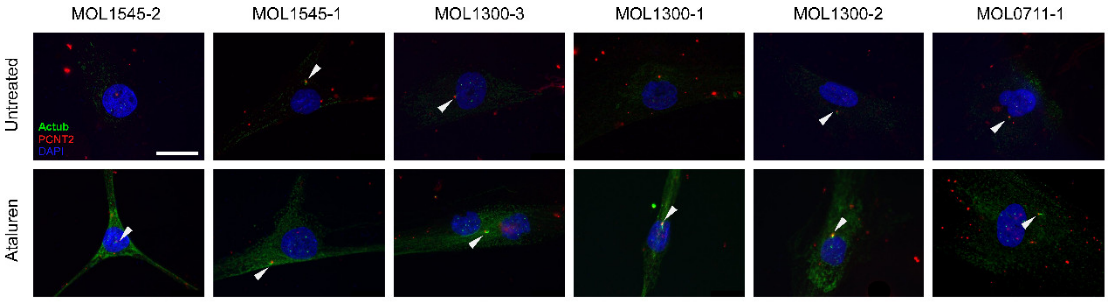

Figure S2. Representative images of fibroblasts of all FAM161A patients participated in this study. Scale bar 25 µm.

Table S1. *In-silico* analysis of predicted amino acid exchanges resulting from translational read-through of p.Arg523*. The following tools were used: SIFT (http://sift.jvic.org), PolyPhen-2 (http://genetics.bwh.harvard.edu/pph2/), and MutationTaster (http://www.mutationtaster.org).

Outcome	***Possible mispairing***	***Amino acid substitution***	***Physical properties***	***SIFT***	***Polyphen-2***	***MutationTaster***
Original	CGA	Arg (R)	Basic<br>Highly polar<br>Positively charged	Healthy	Healthy	Healthy
Nonsense	**T**GA	Termination codon		Damaging [0]	Damaging [1.00]	Disease causing [0.99]
Rescue	**A**GA	Arg (R)	Basic	Healthy	Healthy	Healthy
Missense	**G**GA	Gly (G)	Non-polar	Damaging [0]	Probably damaging [1.00]	Disease causing [0.99]
Nonsense	T**A**A	Termination codon		Damaging [0]	Damaging [1.00]	Disease causing [0.99]
Missense	T**T**A	Leu (L)	Non-polar	Damaging [0]	Probably damaging [1.00]	Disease causing [0.99]
Missense	T**C**A	Ser (S)	Uncharged Polar	Damaging [0]	Probably damaging [1.00]	Disease causing [0.99]
Missense	TG**G**	Trp (W)	Non-polar	Damaging [0]	Probably damaging [1.00]	Disease causing [0.99]
Missense	TG**C**	Cys (C)	Uncharged Polar	Damaging [0]	Probably damaging [1.00]	Disease causing [0.99]
Missense	TG**T**	Cys (C)	Uncharged Polar	Damaging [0]	Probably damaging [1.00]	Disease causing [0.99]

Table S2. Expression of α-tubulin—detailed data for each patient. The number of cells with α-tubulin labelling counted for each cell line are indicated.

		Untr	DMSO	Gent	Ata		Untr	DMSO	Gent	Ata
# of cells counted in total	WT1	300				MOL1545-2	300	300	300	300
# of cells with positive α-tubulin staining		284					253	241	277	284
# of cells counted in total	WT2	300	300	300	300	MOL1545-1	300	300	300	300
# of cells with positive α-tubulin staining		290	283	267	275		254	258	272	282
# of cells counted in total	WT3	300	300	300	300	MOL1300-3	300	300	300	300
# of cells with positive α-tubulin staining		286	285	287	289		262	267	280	288
# of cells counted in total	WT4	300	300	300	300	MOL1300-1	300	300	300	300
# of cells with positive α-tubulin staining		269	272	261	274		245	253	275	279
# of cells counted in total	WT5	300	300	300	300	MOL1300-2	300	300	300	300
# of cells with positive α-tubulin staining		277	269	258	262		269	271	278	281
# of cells counted in total						MOL0711-1	300	300	300	300
# of cells with positive α-tubulin staining							244	227	266	272

Table S3. Summary of ciliogenesis and cilia length analyses—detailed data for each patient. The number of the cells that were counted per each cell line and the number of the ciliated cells that were counted in each experiment. Ata: Ataluren; Gent: Gentamicin; Untr: untreated.

		Untr	DMSO	Gent	Ata		Untr	DMSO	Gent	Ata
# of cells counted in total	WT1	300				MOL1545-2	300	300	300	300
# of ciliated cells		225					121	116	136	231
# of cells counted in total	WT2	300	300	300	300	MOL1545-1	300	300	300	300
# of ciliated cells		244	247	233	242		107	111	115	256
# of cells counted in total	WT3	300	300	300	300	MOL1300-3	300	300	300	300
# of ciliated cells		227	225	237	238		81	92	91	162
# of cells counted in total	WT4	300	300	300	300	MOL1300-1	300	300	300	300
# of ciliated cells		209	247	232	244		121	126	125	188
# of cells counted in total	WT5	300	300	300	300	MOL1300-2	300	300	300	300
# of ciliated cells		202	204	188	204		108	93	101	191
# of cells counted in total						MOL0711-1	300	300	300	300
# of ciliated cells							79	71	82	171
